# Respiratory Muscle

**Published:** 1857-12

**Authors:** 


*Respiratory Muscle.* Observed by POWHATAN JORDAN, M. D., of Washington, D. C.

On the night of the 13th December, 1855, while assisting a student in the dissecting room of the medical department of the Georgetown College, in dissecting out the pectoralis major, I perceived fibres which crossed the general course of the muscle at right angles. Never having seen anything of the kind before, I traced them carefully up, and to my surprise, and to that of the demonstrator, and of the students present, I was able to exhibit, from origin to insertion, a very beautiful triangularly shaped muscle, arising from fifth costal cartilage of the side, by a triangular fleshy belly, becoming tendinous abont two inches from its origin, passing upward obliquely across the sternum, and inserting by a long, fine, bright, silvery tendon into the tendon of the sterno-cleido mastodieus of the left side, or rather in common with that muscle, into the left superior portion of the sternum.

There was no appearance of a corresponding fellow on the opposite side. Its action can be no other than to assist in elevating the costal cartilages, and it therefore must be considered as an accessory muscle of respiration.

Nothing of the kind ever having been seen by any of the faculty of the college, or by myself, at the request of Dr. Johnson Elliot, the professor of anatomy, I removed the sternum with the muscle attached, and made a preparation of it for his cabinet. I should not have thought enough of the matter to have reported it, had it not occurred to me again in the same building, and in this second case, with its fellow of the opposite, fully developed. I was engaged in assisting Dr. Elliot in making a dissection for his next day's lecture, and while displaying the pectoralis major, I remarked muscular fibres similar to those before mentioned. Much struck by the circumstance, I worked them out, and found a muscle in all respects similar to that which I had previously observed. A corresponding muscle in the left side somewhat better developed, arose from fifth and sixth costal cartilages, by a broad, fleshy belly, and about half way between its origin and the top of the sternum, was inserted into the tendon of its fellow, thus making that the common tendon of both muscles. I made a preparation of this, which with the one previously made, were exhibited by Professor Elliot to the class of the medical department of the Georgetown College.

The two preparations were examined by the members of the faculty of the above-named school; also by Dr. Thomas Miller, Professor of Anatomy in the National Medical College, and by Dr. R. K. Stone, formerly Professor of Microscopic Anatomy and of Physiology in the same institution. Neither of these gentlemen had ever before seen anything of the kind, nor could any mention of it be found in Sharpey, Harrison, Horner, Wilson, Bichat, Cruveilhier, or other anatomical authors. It is proper to say, that both subjects were of the African race—the first, a negress of medium size and build, 30 to 35 years old—the second, a negro male of medium height and size, about 45 years of age.

By the advice of Dr. Stone, I showed the preparations to Dr. Baird, the accomplished naturalist of the Smithsonian institution, thinking there might be something similar in some of the lower animals, but he could not say that he had ever seen anything in the brute creation at all corresponding to it.

I am indebted to Dr. Stone for a handsome colored drawing of the preparation, as well as for much assistance in ascertaining the probable originality of the observation.— *Virginia Medical Journal.*

---

*Death after Ligature of Varicose Veins of the Leg.*—We are always anxious to give a prominent place to instances of ill consequences resulting from any of the minor operations usually deemed devoid of risk, since it is very desirable that the profession should be kept well informed respecting them. Ligature of the veins of the leg, when in a varicose state, has recently, in the hands of Messrs. Lee, Erichsen, Partridge, and Simon, been deemed to have almost passed into the category referred to, and has been largely performed, without our having had to record any instances of ill result ensuing. It has been thought that the modified plan of operating now adopted had relieved the procedure of all its danger. Nor are we at all certain that the case we now have to notice is properly to be ranked as a disappointment of these hopes. There are, however, very suspicious circumstances. Although there is no post-mortem evidence of phlebitis or of pyæmic deposits, and although the death followed with such extreme rapidity on the first occurrence of alarming symptoms, and after a severe mental shock had been sustained, yet we have the awkward fact that rigors and vomiting were the first signals of danger. Such instances are, we suspect, not without parallel in the history of pyæmia; those, for instance, in which the patient dies right off in the first shock of the disease, without allowing time for any of the secondary and characteristic phenomena to be developed. Still we doubt not there will be differences of opinion as to the real cause of death in the following case, the brief note respecting which has been kindly supplied to us by the operator:

A woman, aged 44, was admitted on August 4th, into the Westminster Hospital, under the care of Mr. Hillman, on account of varicose veins of the leg, with chronic congestion and superficial ulceration of skin. She was subject to great mental anxiety, but apparently otherwise in good health. The veins were ligatured in four places by hare-lip pins, and over-lying pieces of bougie. The pins were removed on the thirteenth day. Four days after the removal of the pins, when all was going on well, her husband called, and exceedingly distressed her. Rigors and vomiting came on, and she died on the day following his visit.

At the autopsy, no deposition of serum or pus in any organ, or local mischief of any kind was to be detected.—*Ibid.*

